# Thermoelectric Properties of Scandium Sesquitelluride

**DOI:** 10.3390/ma12050734

**Published:** 2019-03-04

**Authors:** Dean Cheikh, Kathleen Lee, Wanyue Peng, Alexandra Zevalkink, Jean-Pierre Fleurial, Sabah K. Bux

**Affiliations:** 1 Thermal Energy Conversion Research and Advancement Group, Jet Propulsion Laboratory/California Institute of Technology, 4800 Oak Grove Drive, Pasadena, CA 91109, USA; dean.a.cheikh@jpl.nasa.gov (D.C.); Kathy.Lee@jpl.nasa.gov (K.L.); Jean-Pierre.Fleurial@jpl.nasa.gov (J.-P.F.); 2 Chemical Engineering and Materials Science Department, Michigan State University, East Lansing, MI 48824, USA; pengwany@msu.edu (W.P.); alexzev@msu.edu (A.Z.)

**Keywords:** thermoelectric, rare-earth telluride, scandium telluride

## Abstract

Rare-earth (RE) tellurides have been studied extensively for use in high-temperature thermoelectric applications. Specifically, lanthanum and praseodymium-based compounds with the Th_3_P_4_ structure type have demonstrated dimensionless thermoelectric figures of merit (*zT*) up to 1.7 at 1200 K. Scandium, while not part of the lanthanide series, is considered a RE element due to its chemical similarity. However, little is known about the thermoelectric properties of the tellurides of scandium. Here, we synthesized scandium sesquitelluride (Sc_2_Te_3_) using a mechanochemical approach and formed sintered compacts through spark plasma sintering (SPS). Temperature-dependent thermoelectric properties were measured from 300–1100 K. Sc_2_Te_3_ exhibited a peak *zT* = 0.3 over the broad range of 500–750 K due to an appreciable power factor and low-lattice thermal conductivity in the mid-temperature range.

## 1. Introduction

Thermoelectric materials are used as solid-state energy conversion devices to transform thermal energy into electrical power. The dimensionless thermoelectric figure of merit is a measure of a material’s conversion efficiency and is defined as $zT=\frac{S^{2}T}{\rho \kappa }$, where *S* is the Seebeck coefficient, *ρ* is the electrical resistivity, *κ* is the thermal conductivity, and *T* is the absolute temperature. Good thermoelectric materials will have a high Seebeck coefficient, low electrical resistivity, and low thermal conductivity.

One area where thermoelectrics are a key enabling technology is power generation for deep-space exploration vehicles [1,2,3]. Radioisotope thermoelectric generators (RTGs) utilize thermoelectric materials to convert heat from a decaying radioisotope fuel into electricity to power spacecraft instrumentation. The thermoelectric materials integrated into the RTGs (traditionally Si–Ge alloys, PbTe, or Te–Ag–Ge–Sb) have demonstrated long-term reliability, with the *Voyager 1* and *2* missions continuously operating for over 40 years [2,3,4,5]. However, one limitation of these devices is that they exhibit modest thermal-to-electrical system level conversion efficiencies of approximately 6.5% at beginning-of-life, due to the average *zT* values of the heritage materials being less than 1 over their operating temperature range [1,4]. Hence, identification of materials with high *zT* values is critical for increasing the efficiencies of RTGs, which would enable greater specific power for future missions.

Rare-earth (RE) tellurides in the range RE_3−x_Te_4_ (0 < *x* < 0.33) have been studied extensively for potential use in high-temperature thermoelectric applications owing to their excellent thermal stability and large *zT* values [6,7,8,9,10,11]. The favorable thermoelectric properties stem from the complexity of the Th_3_P_4_ structure type ($\bar{I}$4*3d*) from vacancies on the RE site, which cause low-lattice thermal conductivities (between 0.5 and 1 $\frac{W}{m\;K}$ for La_3−*x*_Te_4_ and Pr_3−*x*_Te_4_) [2,6,7]. The electronic properties of these compounds are also controlled by the RE vacancies. Applying electron counting rules to RE_3_Te_4_, each RE cation donates 3 electrons and each Te anion accepts 2 electrons. Therefore, when *x* = 0 (RE_3_Te_4_) there is one free electron in the structure, and a degenerate semiconducting behavior is observed. When *x* = 0.33 (RE_2.67_Te_4_ or RE_2_Te_3_), no excess electrons are present and they are semi-insulating. Additionally, these materials have favorable band structures that allow them to maintain large Seebeck coefficients at high carrier concentrations [12,13]. As a result, several RE_3−x_Te_4_ compounds have peak *zT* values over 1, the highest being Pr_3−*x*_Te_4_ with a *zT* = 1.7 at 1200 K for *x* = 0.23 [6,7]. Therefore, it is of interest to investigate other rare-earth tellurides to attain higher figures of merit.

Though not a member of the lanthanide series, scandium is chemically similar to the RE elements [14]. The primary use of scandium is in aluminum alloys for aerospace components, where adding small amounts of scandium significantly improves the specific strength and increases the recrystallization temperatures [15,16,17,18]. Scandium additions to SrCoO_3_-based solid oxide fuel cells have also been shown to significantly increase their oxygen conductivity and improve the overall performance [19,20,21]. Despite the benefits to several applications, the study of scandium-containing compounds has been limited but promising. Doping Mg_2_Si and ZnCdO with scandium has been shown to increase the power factor ($\frac{S^{2}}{\rho }$) significantly [22,23]. Additionally, thin films of ScN are known to have large power factors when compared to other transition metal nitrides [24,25]. Therefore, investigation of other compounds containing scandium is of interest.

Here, we present an investigation of scandium sesquitelluride (Sc_2_Te_3_). While other rare-earth tellurides have been investigated for high-temperature thermoelectric applications, prior studies of Sc_2_Te_3_ were limited to structural studies [26,27]. Unlike other RE_3−x_Te_4_ (RE = La, Ce, Pr, Nd) compounds, Sc_2_Te_3_ does not adopt the Th_3_P_4_ structure type. Men’kov et al. first reported Sc_2_Te_3_ in the cubic (*Fm*$\bar{3}$*m*) structure type, which is a rock salt (NaCl) structure with 1/3 of the Sc sites vacant [26]. White and Dismukes also obtained Sc_2_Te_3_ in a rhombohedral (R$\bar{3}$*m*) structure, which can be described as alternating regions of the NaCl and NiAs structure types [27]. Both phases were produced using a similar method of direct reaction of elements, and the reason for the differences in reported crystal structures is still undetermined. However, these structures are relatively complex and would likely be favorable to have intrinsically low-lattice thermal conductivities (*κ_L_*). Furthermore, point-defect scattering from RE vacancies is known to contribute to the low *κ*_L_ of the Th_3_P_4_ structure, where up to 1/9 of the cation positions may be vacant [6,7]. Similarly, the comparatively larger number of cation vacancies in the cubic Sc_2_Te_3_ structure (1/3 of the Sc positions) may enhance phonon scattering compared to Th_3_P_4_ compounds.

Although Sc_2_Te_3_ has traditionally been made using melt synthesis, we employed a low-temperature mechanochemical synthesis to avoid sample inhomogeneity and compositional variations due to the large differences in melting points of Sc and Te (1541 and 449 °C, respectively) [6,7]. After synthesis, the compound was compacted using spark plasma sintering (SPS) and characterized using powder X-ray diffraction (XRD) and scanning electron microscopy (SEM). The high-temperature thermoelectric properties (*ρ*, *S*, and *κ*) were measured up to 1100 K, and *zT* was calculated.

## 2. Materials and Methods

### 2.1. Synthesis

Sc_2_Te_3_ was synthesized using a mechanochemical approach. Elemental Sc (99.9%, Chemistry Cabinet) and Te shot (99.999%, 5N Plus) were combined in an argon-filled glovebox and placed in a stainless-steel ball mill vial with stainless-steel balls. It was then ball milled (SPEX SamplePrep 8000, SPEX SamplePrep, Metuchen, NJ, USA) for over 10 h until a homogenous, black powder of Sc_2_Te_3_ was produced. The powder was then compacted in a 12.7-mm graphite die using spark plasma sintering (SPS) at a temperature above 1450 K and a pressure of 80 MPa for 30 min under vacuum. From geometric measurements, the ingot was found to have a density of 99% the theoretical value (Table S1). A sample was then cut from the ingot using a diamond saw and ground to a thickness of approximately 1 mm for high-temperature measurements.

### 2.2. Characterization

Powder X-ray diffraction (XRD) data was collected with a Phillips PANalytical X’Pert Pro diffractometer (Philips PANalytical, Westborough, MA, USA) using Cu Kα radiation. Due to the Sc_2_Te_3_ powder being highly air sensitive, the powders were sealed with 1-mm-thick Kapton film on a Si zero background holder under argon. Compacted samples were ground using a mortar and pestle, and scans were performed over a 2*θ* range of 20°–70°, with a 0.02° step size and a time of 487 s per step. Back-scattered electron (BSE) scanning electron microscope (SEM) images were taken on a Zeiss 1550 VP SEM (Carl Zeiss AG, Oberkochen, Germany).

Once compacted, the sample was stable enough to be handled outside the glovebox; however, a native oxide would form slowly if left out for extended periods (>10 min). Due to this, the sample surface was polished immediately before measurements were taken. A custom-built combined 4-point probe and Hall effect system was used to measure the electrical resistivity and Hall voltage, from which the carrier concentration and Hall mobility were calculated [28]. The Seebeck coefficient was measured using a custom-fabricated instrument [29]. The specific heat capacity was measured using a Netzsch DSC 404 (Netzsch, Selb, Germany), and thermal diffusivity was measured using a Netzsch LFA 457 system (Netzsch, Selb, Germany). The thermal conductivity was calculated by *κ* = *DC_p_d* where *κ* is the thermal conductivity, *D* is the thermal diffusivity, *C_p_* is the specific heat capacity, and *d* is the sample density. The measured *C_p_* is shown in Figure S1.

The temperature-dependent elastic moduli of Sc_2_Te_3_ was measured by resonant ultrasound spectroscopy (RUS) under flowing Ar using a modified Magnaflux-RUS Quasar 4000 system (Magaflux, Glenview, IL, USA) [30,31]. Buffer-rods were glued to the transducer that extended into the furnace for sample mounting. Cylindrical samples were mounted on a tripod transducer setup, where one transducer induced vibrations and the remaining two detected the specimen resonances. The sinusoidal driving frequency was swept from 0 to 500 kHz. The elastic moduli were measured in 20 K intervals from 303 K up to 673 K. The data was analyzed using commercial Quasar2000 CylModel software (Version 2.68b, Magaflux, Glenview, IL, USA) to match the observed and predicted resonant frequencies peaks. At room temperature, 19 peaks were fitted, yielding a standard deviation of 0.09%. Since the elastic tensor value does not change after fitting the first 6 peaks, only the first 6 peaks were used for high-temperature analysis.

High-temperature X-ray diffraction of Sc_2_Te_3_ was measured from 303 K to 553K in 20 K intervals using a Rigaku Smartlab XRD system (Rigaku, The Woodlands, TX, USA) (Cu K_α_ radiation) equipped with a high-temperature stage. The sample was ground into a fine powder and placed on graphite foil on top of a platinum tray. The measurement was performed under vacuum (10^−4^ Torr) to prevent oxidation. The thermocouple was in contact with the inner part of the platinum tray to increase the accuracy of the temperature measurement. A heating rate of 10 K/min was used with a 1-min hold, and sample height alignments were performed before each measurement to account for the thermal expansion of the holder and sample. Phase purity of the samples was confirmed via peak matching within the ICSD database, and lattice parameters were obtained using peak indexing using PDXL2.

## 3. Results and Discussion

### 3.1. Phase Analysis

**Figure 1:**
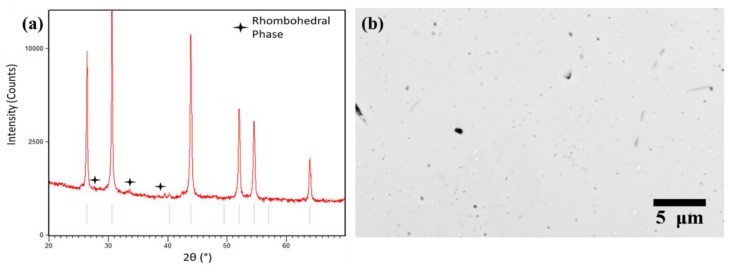
(**a**) Powder X-ray diffraction pattern of Sc_2_Te_3_ sample. The pattern agrees well with the Sc_2_Te_3_ phase (blue lines). A small amount of the rhombohedral Sc_2_Te_3_ phase was detected and marked. (**b**) Back-scattered electron (BSE) SEM micrograph of the polished surface of the Sc_2_Te_3_. Dark regions are from residual porosity present in the sample. A small number of Fe contaminants (light regions) were observed and confirmed through energy dispersive X-ray spectroscopy (EDS).

To verify the phase of the sample, a densified compact was ground using a mortar and pestle and analyzed using powder X-ray diffraction (Figure 1a). The pattern correlated with the cubic (*Fm*$\bar{3}$*m*) Sc_2_Te_3_ structure [26]. A small amount of the rhombohedral phase (*R*$\bar{3}$*m*) was detected. The homogeneity of the sample was further verified by back-scattered electron (BSE) scanning electron microscopy (SEM) (Figure 1b). The uniform contrast reflects the phase homogeneity of the sample. Energy dispersive X-ray spectroscopy (EDS) also confirmed an even distribution of Sc and Te throughout the sample (Figure S1), with dark regions representing residual porosity in the sample. A small number of Fe inclusions were also observed, likely coming from the vials used during ball milling. The measured room temperature lattice parameter value of *a* = 5.831 ± 0.005 Å was in excellent agreement with prior literature (5.817 Å) [26].

### 3.2. Electronic Transport Properties

**Figure 2:**
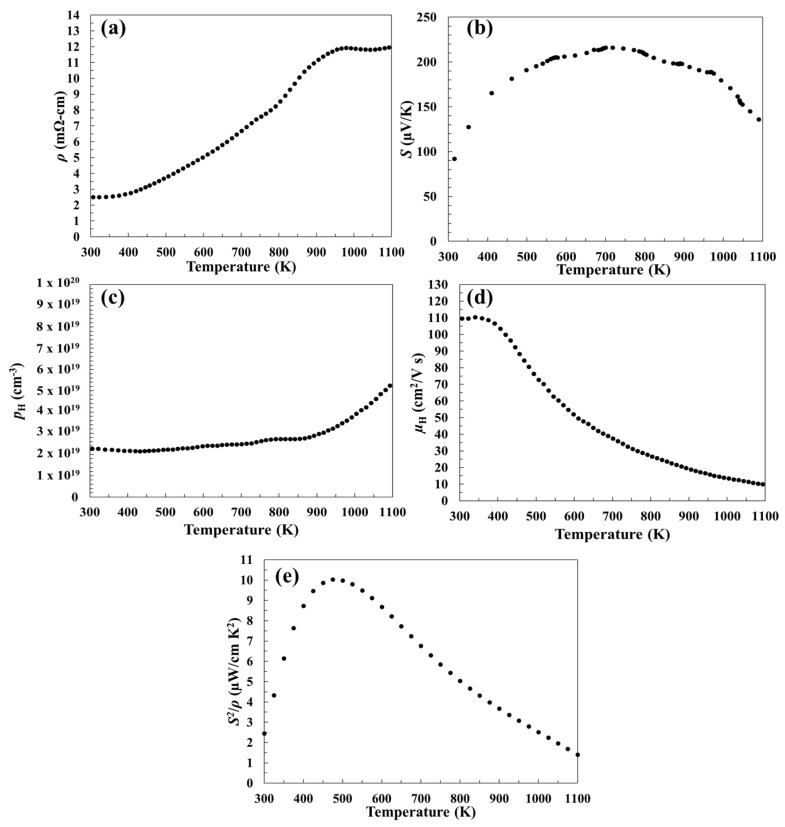
Temperature-dependent (**a**) electrical resistivity, (**b**) Seebeck coefficient, (**c**) Hall carrier concentration, (**d**) Hall mobility, and (**e**) power factor of Sc_2_Te_3_.

The high-temperature electrical resistivity (*ρ*) of Sc_2_Te_3_ is shown in Figure 2a. The *ρ* increases with increasing temperature, as expected for a degenerate semiconductor. The Seebeck coefficient (*S*) (Figure 2b) was positive, indicating *p*-type conduction, and was relatively constant from 500–1000 K. The positive *S* and Hall carrier concentration (Figure 2c) suggest that the sample is slightly Te-rich, relative to the nominal, charge-balanced Sc_2_Te_3_ composition.

Above 1000 K, a decrease in *S* and a change in the slope of *ρ* were observed. Both of these correspond to an increase in the Hall carrier concentration (*p*_H_), shown in Figure 2c, indicating minority carrier activation. The Hall mobility (Figure 2d) decreased with increasing temperature, which is expected for acoustic phonon scattering. The decreasing mobility accounts for the increase in resistivity until 900 K, above which the increase in carrier concentration leads to a plateau in *ρ*. The thermal bandgap (*E_g_*) was calculated to be 0.31 eV using the approximation *S* = *E_g_*/2*eT_max_*, where *T_max_* is the temperature where the maximum value of *S* occurs [32]. This value is lower than the bandgaps for other rare-earth tellurides (approximately 0.9 eV for La_3−x_Te_4_ and Pr_3−x_Te_4_) [6,7]. The power factor (*S*^2^/*ρ*) was calculated by combining *S* and *ρ* (Figure 2e). Due to the sharp increase in *S* at low temperatures, an appreciable power factor is achieved from 400 to 600 K. However, at higher temperatures *S*^2^/*ρ* decreases from the increased resistivity and reduction in Seebeck.

### 3.3. Thermal Transport Properties

**Figure 3:**
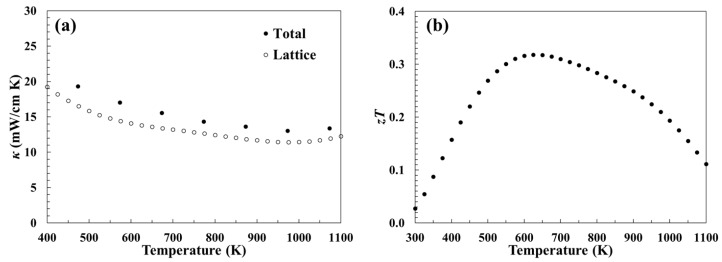
Temperature-dependent (**a**) thermal conductivity and (**b**) thermoelectric figure of merit.

The total thermal conductivity (filled circles in Figure 3a) was found to range from 15 to 22 mW·cm^−1^·K^−1^, similar to that of other rare-earth tellurides [6,7]. The lattice thermal conductivity (*κ_L_*) was determined by subtracting the electronic contribution (*κ_e_*) from the total thermal conductivity, where *κ_e_* was calculated using the Weidemann–Franz law (*κ_e_* = *LT*/*ρ*). A simplified temperature-dependent Lorenz number (*L*) was calculated as a function of Seebeck coefficient using the estimation *L* = 1.5 + exp(−|*S*|/116), resulting in values between 1.7 and 2.2 × 10^21^ W·Ω·K^−2^ [33]. This approximation is calculated from a single parabolic band model and assumes acoustic phonon scattering of charge carriers. *κ_L_* was the primary contributor to the total thermal conductivity (Figure 3a, open circles) with values between 11 and 18 mW·cm^−1^·K^−1^.

**Figure 4:**
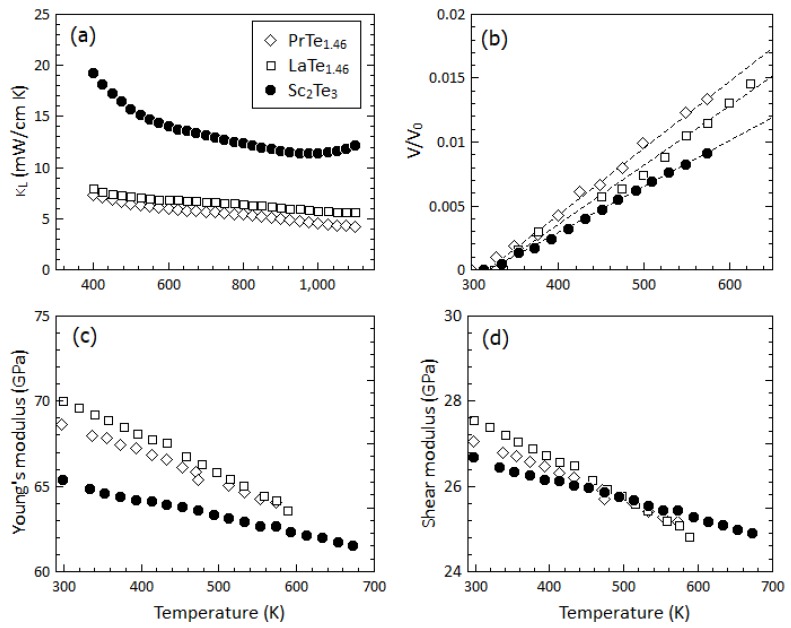
A comparison of (**a**) the lattice thermal conductivity, (**b**) experimental volume thermal expansion, *V*/*V_o_* (**c**) the temperature-dependent Young’s moduli, and (**d**) shear moduli of PrTe_1.46_, LaTe_1.46_, and Sc_2_Te_3_. Data for PrTe_1.33_ and LaTe_1.46_ are from reference [7].

As shown in Figure 4a, the *κ_L_* of Sc_2_Te_3_ is significantly higher than that of Pr_3−*x*_Te_4_ or La_3−*x*_Te_4_ [7]. To explain the disparity in *κ_L_*, several factors must be considered. Both structure types contain a large concentration of vacancies on the cation site (30% in Sc_2_Te_3_ and 11% in the RE_3−x_Te_4_ samples highlighted here), which leads to significant scattering of high-frequency phonons in all three cases. We also consider differences in the speed of sound and Grüneisen parameters, evaluated by means of the temperature-dependent elastic moduli and the thermal expansion behavior shown in Figure 4. Resonant ultrasound spectroscopy (RUS) was performed up to 673 K to determine the temperature-dependent Young’s (Y) and shear (G) moduli. Although the elastic moduli of Sc_2_Te_3_ at room temperature are actually softer than those reported for La_3−x_Te_4_ and Pr_3−x_Te_4_, the lower theoretical density of Sc_2_Te_3_ leads to slightly higher longitudinal and transverse speeds of sound (see Table 1) [7]. We also find that Sc_2_Te_3_ exhibits a lower thermal expansion coefficient and a lower bond softening rate than RE_3−x_Te_4_ (RE = Pr or La) samples (see Figure 4b–d). This is indicative of a lower Grüneisen parameter in Sc_2_Te_3_, possibly due to the smaller coordination numbers of the cation site (6-fold for Sc versus 8-fold for La and Pr) [34,35]. Indeed, estimating the average thermodynamic Grüneisen parameter as *γ* = *αB*/*C_υ_ρ* (*α* is the volume thermal expansion coefficient, *B* is the bulk modulus, *C_υ_* is the heat capacity, and *ρ* is the theoretical density) yields much lower values for Sc_2_Te_3_, which would lead to lower rates of phonon–phonon scattering. Thus, the combination of a slightly higher sound velocity and lower Grüneisen parameter in Sc_2_Te_3_ is likely the origin of its higher *κ_L_* in the measured temperature range.

### 3.4. Dimensionless Thermoelectric Figure of Merit

The thermal and electronic transport properties were used to calculate the dimensionless thermoelectric figure of merit (*zT*) (Figure 3b). The sharp rise in the power factor at low temperatures allows for Sc_2_Te_3_ to attain a maximum *zT* value of 0.3 from 500 to 750 K. Additionally, the relative flatness of the Seebeck coefficient with respect to temperature causes *zT* to maintain values close 0.3 over the mid-temperature range. This peak thermoelectric performance at intermediate temperatures contrasts the behavior of other rare-earth tellurides, where their properties optimize in the high-temperature regime (>1200 K) [2,6,7].

## 4. Conclusions

Sc_2_Te_3_ was successfully synthesized using a mechanochemical approach and compacted using spark plasma sintering. Powder XRD confirmed the samples were Sc_2_Te_3_ with the space group *Fm*$\bar{3}$*m*. The temperature-dependent electronic and thermal transport properties were measured. The power factor was found to achieve values near 10 µW/m·K^2^ in the intermediate-temperature range due to a large, *p*-type Seebeck coefficient. *κ_L_* was determined to be the dominant contribution to the thermal conductivity. Compared with RE_3−x_Te_4_ (RE = Pr or La) compounds, the *κ_L_* of Sc_2_Te_3_ was higher due to its higher speed of sound and lower Grüneisen parameter. A *zT* value of 0.3 was achieved in the mid-temperature range of 500–750 K. Modification to the carrier concentration or addition of dopants for point-defect scattering may help improve the properties of Sc_2_Te_3_.

## Figures and Tables

**Table 1:** Elastic moduli, speed of sound, density, volumetric coefficient of thermal expansion, and Grüneisen parameters for Sc_2_Te_3_ compared with PrTe_1.46_ and LaTe_1.46_ [7].

Parameters	PrTe_1.46_	LaTe_1.46_	Sc_2_Te_3_
Young’s modulus, *Y* (GPa)	68.6	70.1	65.4
Shear modulus, *G* (GPa)	27.1	27.6	26.7
Bulk modulus, *B* (GPa)	49.3	50.9	39.6
Long melocity, *v_L_* (m/s)	3543	3642	3773
Transverse velocity, *v_T_* (m/s)	1994	2042	2247
Density, *ρ* (g/cm^3^)	6.804	6.607	5.285
Volume CTE (10^−5^/K)	5.06	4.62	3.60
Grüneisen parameter, *γ*	1.96	1.89	1.03

## Supplementary Materials

The following are available online at https://www.mdpi.com/1996-1944/12/5/734/s1. Figure S1. Temperature-dependent heat capacity. Table S1. Sample density measurements.
