# Supplementary material for: Thermoelectric Properties of Scandium Sesquitelluride
Source: Materials (Basel). 2019 Mar 4;12(5):734. doi: 10.3390/ma12050734 (PMC6427805; doi:10.3390/ma12050734)
Supplement: Supplementary file 1 [file materials-12-00734-s001.pdf]

materials

Supplementary Information

Thermoelectric Properties of Scandium Sesquitelluride

Dean Cheikh [1], Kathleen Lee [1], Wanyue Peng [2], Alexandra Zevalkink [2], Jean-Pierre Fleurial [1] and Sabah K. Bux [1,*]

[1] Thermal Energy Conversion Research and Advancement Group, Jet Propulsion Laboratory/California Institute of Technology, 4800 Oak Grove Drive, Pasadena, CA 91109, USA; dean.a.cheikh@jpl.nasa.gov (D.C.); Kathy.Lee@jpl.nasa.gov (K.L.); Jean-Pierre.Fleurial@jpl.nasa.gov (J.-P.F.)

[2] Chemical Engineering and Materials Science Department, Michigan State University, East Lansing, MI 48824, USA; pengwany@msu.edu (W.P.); alexzev@msu.edu (A.Z.)

* Correspondence: Sabah.K.Bux@jpl.nasa.gov; Tel.: +1-818-393-7067

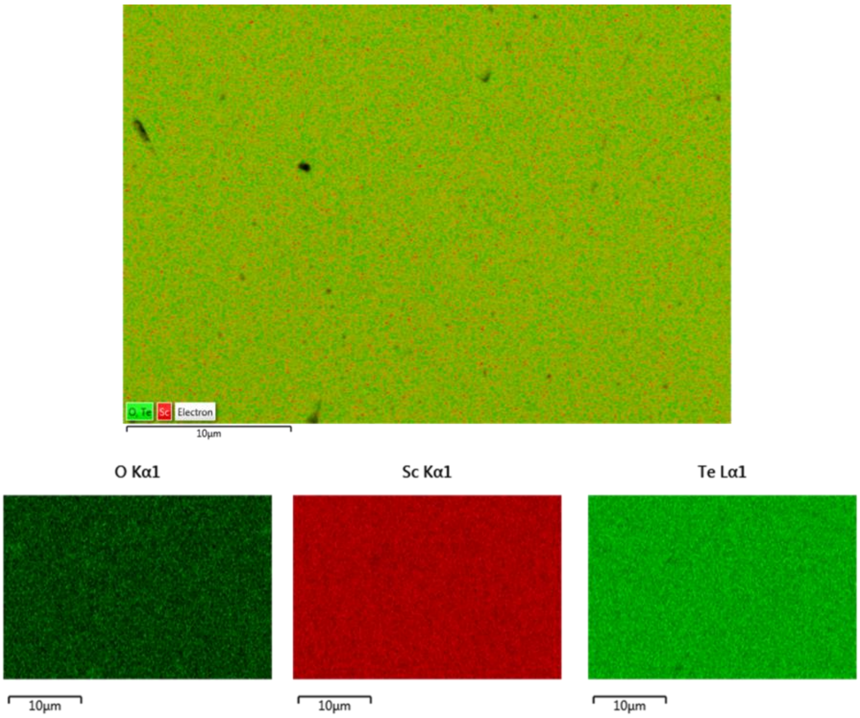

Figure 1. EDS map of compacted Sc_2Te_3 sample. Sc and Te were found to be evenly distributed. The detected O resulted from surface oxidation.

Table 1. Measured and theoretical sample densities for Sc_2Te_3. Measurements were made using sample geometry. The sample was over 99% dense.

Sample Density	Theoretical Density
5.29 g/cm^3	5.32 g/cm^3
